# Salt Spray Resistance of Roller-Compacted Concrete with Surface Coatings

**DOI:** 10.3390/ma16227134

**Published:** 2023-11-12

**Authors:** Huigui Zhang, Wuman Zhang, Yanfei Meng

**Affiliations:** School of Transportation Science and Engineering, Beihang Univerisity, Beijing 100191, China; sy1913202@buaa.edu.cn (H.Z.); sy2013201@buaa.edu.cn (Y.M.)

**Keywords:** salt spray, wear resistance, impact resistance, surface coating, roller-compacted concrete (RCC)

## Abstract

In order to evaluate the feasibility of surface coatings in improving the performance of RCC under salt spray conditions, sodium silicate (SS), isooctyl triethoxy silane (IOTS), and polyurea (PUA) were used as surface coatings to prepare four types of roller-compacted concrete (RCC): reference RCC, RCC-SS, RCC-IOTS, and RCC-PUA. A 5% sodium sulfate solution was used to simulate a corrosive marine environment with high temperatures, high humidity, and high concentrations of salt spray. This study focuses on investigating various properties, including water absorption, abrasion loss, compressive strength, dynamic elastic modulus, and impact resistance. Compared to the reference RCC, the 24 h water absorption of RCC-SS, RCC-IOTS, and RCC-PUA without salt spray exposure decreased by 22.8%, 77.2%, and 89.8%, respectively. After 300 cycles of salt spray, the abrasion loss of RCC-SS, RCC-IOTS, and RCC-PUA reduced by 0.3%, 4.4%, and 34.3%, respectively. Additionally, their compressive strengths increased by 3.8%, 0.89%, and 0.22%, and the total absorbed energy at fracture increased by 64.8%, 53.2%, and 50.1%, respectively. The results of the study may provide a reference for the selection of coating materials under conditions similar to those in this study.

## 1. Introduction

The service life of marine concrete structures is contingent upon concrete durability, often impacted by environmental factors, particularly in harsh conditions [1,2]. The salinity-induced corrosion prevalent in marine environments poses a significant threat to critical infrastructure [3,4,5]. To address durability issues arising from scouring and the multi-factor coupling in marine concrete, researchers focus on two aspects: enhancing permeability resistance through admixture additions [6] and bolstering durability via the application of surface coatings [7]. These measures aim to extend the service life of marine concrete structures.

Concrete surface coatings, essential for protecting structures in corrosive environments [7,8,9], fall into three main categories: organic coatings, inorganic coatings, and organic–inorganic composite coatings, depending on their chemical composition [10,11]. Organic coatings primarily comprise polymers like polyacrylate, epoxy resin, polyurethane, and fluorine resin [12,13]. Inorganic coatings include water-soluble silicates, silica sol, phosphates, and more. The growing attention to organic–inorganic composite coatings stems from their flexible composition and synergistic performance benefits. Polymer cement-based coatings, representing this category, demonstrate commendable mechanical properties, corrosion resistance, and weather resistance.

Mehdi et al. [14]. identified epoxy polyurethane and aliphatic acrylic as the most effective coatings for reducing chloride ion penetration and extending the service life of concrete structures. Almusallam et al. [15] compared the durability of epoxy- and polyurethane-coated concrete to that of acrylic, polymer, and chlorinated rubber coatings, finding the former to be superior. Elnaggar et al. [16] developed asphaltic polyurethane coatings with varying NCO/OH ratios, demonstrating high performance in aggressive environments. The Center for Innovative Grouting Materials and Technology devised tests and analytical models to evaluate epoxy- and polyurethane-coated concrete performance [17].

Additionally, Shi et al. [18] observed that polymer coatings enhanced the resistance of surface layer concrete to chloride ion diffusion. Maj and Ubysz [19] investigated the factors contributing to the loss of adhesion of polyurea coatings to concrete substrates in chemically aggressive water tanks. Santos et al. [20] proposed polyurea coatings as a retrofit option for non-load-bearing concrete masonry walls. Arabzadeh et al. [21] assessed superhydrophobic nanomaterial-based coatings on concrete surfaces for water repellency. Yin et al. [22] developed superhydrophobic coatings based on bionic mineralization to enhance marine concrete durability. Moon et al. [23] reported that calcium–silicate compound coatings improved resistance to chloride penetration, freezing–thawing, and carbonation in concrete specimens. Luo et al. [10] integrated kaolinite nanosheets into permeable epoxy resin, resulting in a high-adhesion, barrier-performance organic–inorganic composite coating. Li et al. [24] enhanced the waterproofing and chloride resistance of concrete by designing a nano-polymer-modified cementitious coating, incorporating nano-SiO_2_ or nano-TiO_2_ suspensions into an acrylic emulsion.

The cost-effectiveness and environmentally friendly nature of surface coatings have led to widespread use in various protective engineering applications [25,26]. However, the variety of surface coatings complicates the selection process. Even with similar generic chemical compositions, these coatings offer varying levels of protection, making the right choice challenging [27,28]. In addition, the environmental conditions in the island salt spray zone are different from those in the tidal and submerged zones [29]. In the South China Sea, the average annual temperature is as high as 28.6 °C, and the road surface temperature is as high as 60 °C in summer. The salinity of surface seawater ranges from 33.0 to 33.5; thus, the islands are characterized by high temperatures, high humidity, and high concentrations of salt spray.

In this study, sodium silicate (SS), isooctyl triethoxy silane (IOTS), and polyurea (PUA) were used as the surface coatings. Roller-compacted concrete (RCC), commonly used for airport runways, was prepared with and without a surface coating. Since the abrasion resistance and impact resistance requirements of the airport runways are higher than those of other ordinary building structures, these two properties of RCC, exposed to salt spray, were tested in this study. The microstructures and pore size distribution were also measured. The objective of this study was to evaluate the feasibility of surface coatings in improving the performance of RCC used in island airport runways under salt spray conditions.

## 2. Experimental Produce

### 2.1. Raw Materials

The chemical composition of 42.5-grade Portland cement was detailed in Table 1, with river sand’s fineness modulus specified as 2.43 and coarse aggregate exhibiting a particle size range of 5–15 mm. Workability enhancement utilized a water-reduction agent. RCC mix proportions, computed following GJB 1578-1992 [30], are presented in Table 2. Surface coatings encompassed sodium silicate (SS), isooctyl triethoxy silane (IOTS), and polyurea (PUA) (see Table 3).

### 2.2. Samples Preparation

The size of the prismatic specimen was 100 mm × 100 mm × 400 mm, and the side length of the cubic specimen was 150 mm. Concrete mixture was poured into the test molds in three layers, and each layer was compacted with a vibrating hammer for 30 s. After 24 h, the specimens were demolded and cured under the standard conditions.

The treatment process of the coating materials was carried out in accordance with the manufacturer’s recommendations for use.

In the case of treating RCC with sodium silicate (RCC-SS), the process occurs at 7 days of curing. After removing the specimens from the curing room, their surfaces were brushed with a wire brush. Subsequently, sodium silicate, dissolved in warm water, was evenly sprayed onto the specimen surfaces. This treatment was repeated every two hours for a total of four applications. Finally, the treated specimens were returned to the standard curing chamber and allowed to cure until reaching 28 days.

For RCC treated with isooctyl triethoxy silane (RCC-IOTS), the process was initiated at 28 days of curing. After removing the specimens from the curing room, their surfaces were brushed with a wire brush. Isooctyl triethoxy silane was sprayed onto the specimen surfaces and left for 6 h before a second round of spraying was conducted.

In the case of RCC treated with polyurea materials (RCC-PUA), components A and B were mixed in a ratio of 1:0.45, and a specified amount of butyl acetate was added as a diluent. The treatment was also carried out at 28 days of curing. After removing the specimens from the curing room and brushing their surfaces with a wire brush, the polyurea mixture was uniformly sprayed onto the specimen surfaces. The specimens were then left at room temperature until the polyurea mixture hardened.

In this study, there was one group of reference specimens (without surface treatment) and three groups of surface-treated specimens, which were RCC-SS, RCC-IOTS, and RCC-PUA. The above four groups of specimens were subjected to the performance tests mentioned below, and each group of specimens contained three specimens. The average of the test results of the three specimens was used for comparative analysis. 

Cubic specimens are used in the water-absorption tests, mass change, and abrasion tests. Prismatic specimens are used in the dynamic elastic modulus and impact tests. The compressive strength of the specimen is determined by using the prisms that break in the impact test, and a 100 mm × 100 mm × 10 mm steel plate is placed on the upper and lower surfaces, respectively, along the length of the specimen, so that the area of the compression surface of the specimen is 100 mm × 100 mm.

### 2.3. Salt Spray Cycles

**Figure 1:**
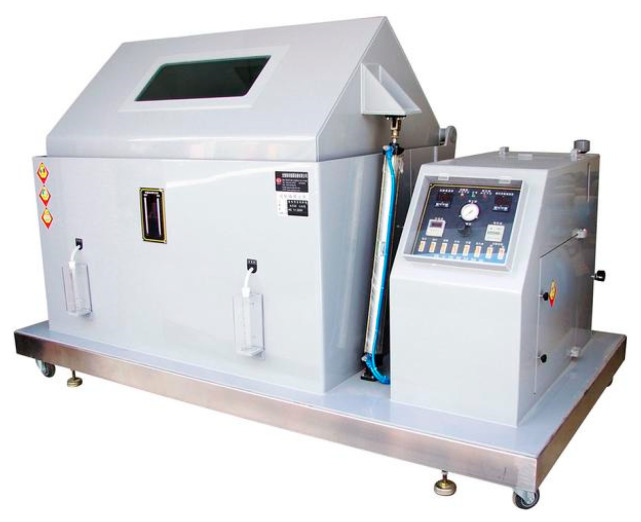
Automatic machine for cyclic exposure to salt spray conditions.

Salt spray conditions in the South China Sea were simulated via indoor salt spray tests. RCC underwent corrosion tests in an automatic machine (see Figure 1) following GB10125-1997 [31] for cyclic exposure to salt spray conditions. A 5% Na_2_SO_4_ (*w*/*w*) was used for salt spray, and the deposition rate was 1.2 mL/(80 cm^2^·h). After the specimen reached the age of maintenance, the specimen was uniformly arranged in the specimen holder to ensure that the upper surface of the specimen was horizontal and the interval between the specimens was more than 100 mm. Each cycle started with 4 h of salt spray at 26.5 °C with 96% humidity, followed by 2 h of drying conditions without salt spray at 50 °C. In order to minimize the effect of salt spray inhomogeneity on the results, the left, right, front, and rear specimens were repositioned every 5 salt spray cycles.

### 2.4. Abrasion Resistance

**Figure 2:**
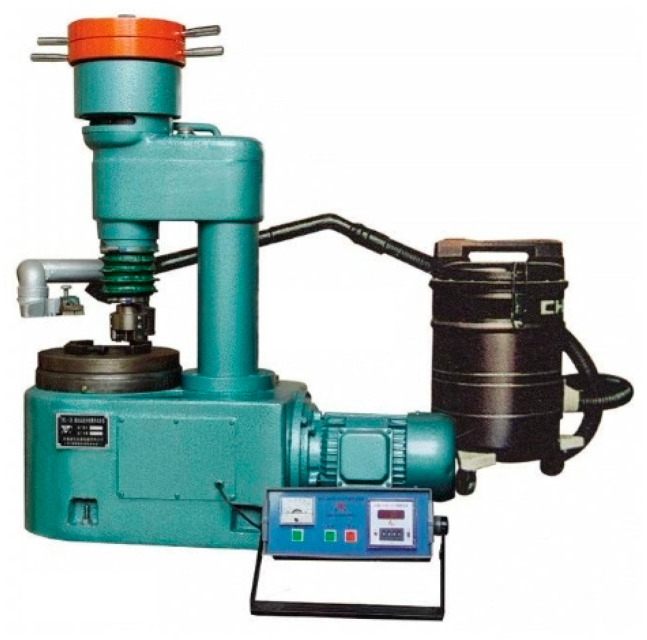
Abrasion test machine.

Abrasion resistance, assessed using JTG E30-2020 [32], was measured with a 200 N load on the pressure head. The horizontal pallet rotated at a speed of 17.5 r/min, with a transmission ratio of 35:1 between the pallet and the spindle (see Figure 2). Abrasion resistance was evaluated based on mass loss per unit area.

### 2.5. Impact Resistance

**Figure 3:**
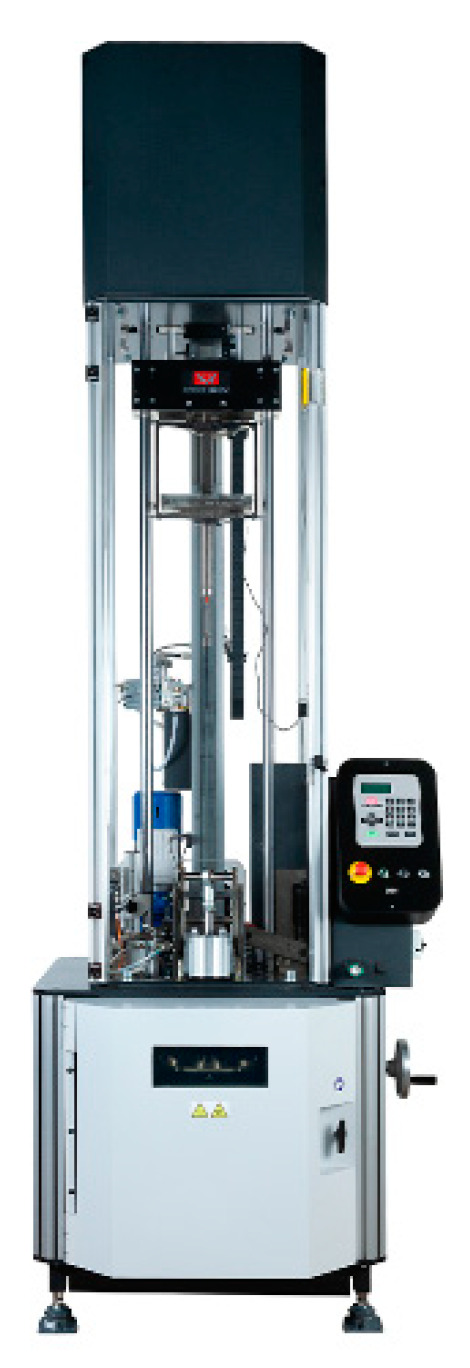
Drop hammer impact tester.

For impact resistance testing, the study employed a drop hammer impact tester (INSTRON 9350 HV, Norwood, MA, USA; see Figure 3). The tester, featuring a square shape with a 75 mm diameter circular area at the center and a double-layer pneumatic clamp, was used. During the impact test, the crosshead, holding the drop hammer, was released, allowing it to fall vertically along two guide frames and impact the specimen within the circular area. A sensor automatically recorded load, displacement, and time data to monitor changes. Simultaneously, a computer data acquisition system integrated the force–displacement curve to determine variations in impact energy absorbed by the specimen. The test utilized a hemispherical indenter measuring 12.6 mm in diameter and weighing 12.250 kg, adjusting the indenter’s height to achieve initial impact energies.

## 3. Results and Discussion

### 3.1. Water Absorption of RCC without Salt Spray Cycles

Water absorption stands as a crucial transport property of concrete, as it serves as the primary avenue for the infiltration of aggressive ions. This penetration through water absorption is a key contributor to durability-related damage and the subsequent degradation of performance. While diffusion does contribute to ionic transport, studies indicate that individual diffusion is a notably slow process. Consequently, water absorption emerges as the dominant mechanism. Theoretically, considering its prevalence, water absorption can be viewed as a representative descriptor that effectively mirrors the durability of concrete [33].

**Figure 4:**
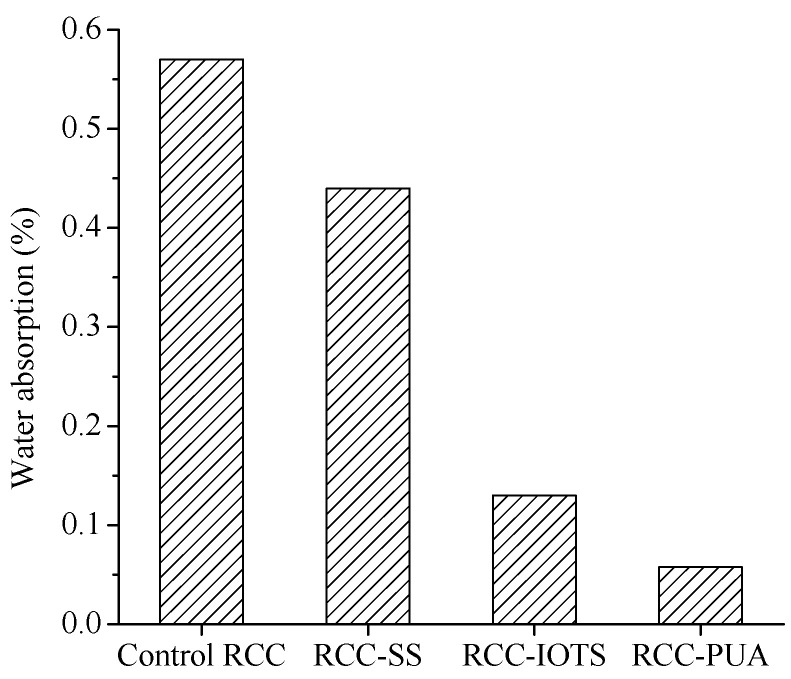
Water absorption of RCC.

**Figure 5:**
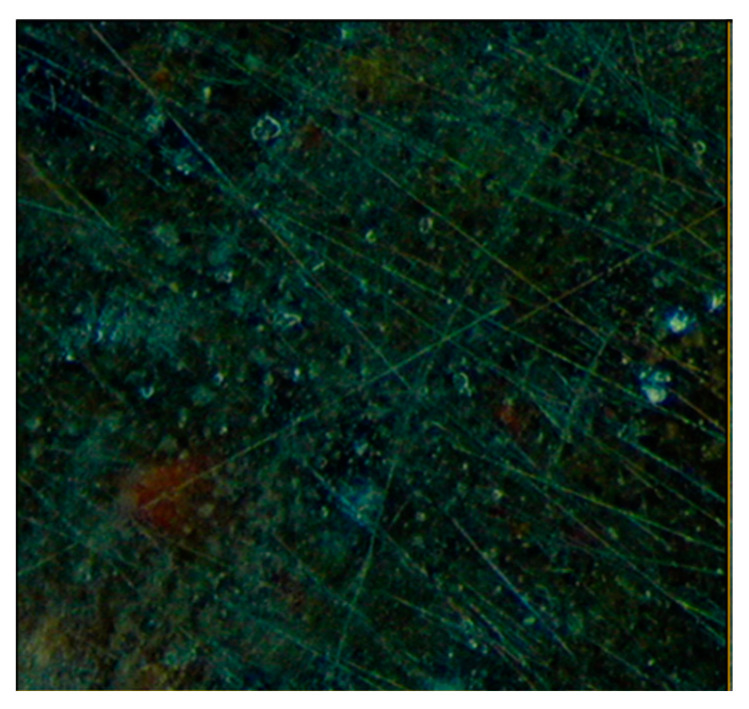
Surface of RCC-PUA.

The water absorption of RCC at 24 h is presented in Figure 4. The water absorption of RCC-SS, RCC-IOTS, and RCC-PUA surface coatings decreased by 22.8%, 77.2%, and 89.8%, respectively. RCC with surface coatings had significantly reduced water absorption compared to RCC without surface coatings. This indicates that the three coating materials effectively act as barriers, thereby significantly reducing the water permeability of RCC. In particular, the PUA surface coating forms a complete, smooth, and dense isolation layer on the surface of RCC (see Figure 5). Therefore, among the three surface coating materials, RCC with PUA surface coating exhibits the lowest water-absorption rate.

Franzoni et al. [34] noted that concrete treated with SS demonstrated approximately half the 7-day water absorption compared to untreated samples. Almusallam et al. [15] and Zhu et al. [35] also highlighted that surface treatment enhanced resistance to capillary water absorption. The findings of this study are consistent with the above results. Moreover, the water absorption of concrete is directly linked to its impermeability, with lower water absorption typically indicating excellent impermeability. These outcomes are consistent with Mehdi et al.’s [14] discovery that PUA surface coating reduced chloride ion diffusion.

### 3.2. Mass Change of Specimen

In a sodium sulfate environment, excessive crystal formation during subsequent salt spray cycles imposes expansive stress on the cement matrix. This stress, in turn, induces the development of micro-cracks and a decline in compressive strength [36]. Beyond osmotic and crystallization pressures, Na_2_SO_4_ exhibits a reversible transformation between its dehydrated and anhydrous states. Studies indicate that Na_2_SO_4_ can generate pore pressures ranging from 400 to 5000 psi, whereas Na_2_SO_4_·10H_2_O can induce pore pressures of 1000–1200 psi [37].

Furthermore, sulfate ions react with the hydration products of cement to produce new products that expand in volume, such as gypsum and ettringite (see Equations (1) and (2)). The volume expansion of ettringite leads to the expansion of existing cracks and the creation of new cracks in the concrete, ultimately reducing the strength of the concrete under sulfate attack [36,38].

Na_2_SO_4_ + Ca(OH)_2_ + 2H_2_O→ CaSO_4_·2H_2_O + 2NaOH (1)

C-A-H + 3CaSO_4_·2H_2_O + 2H_2_O→ C_3_A·3CaSO_4_·32H_2_O (2)

The initial physical sulfate attacks tend to augment the weight of concrete. However, subsequent physical and chemical assaults typically lead to concrete cracking and mass loss.

**Figure 6:**
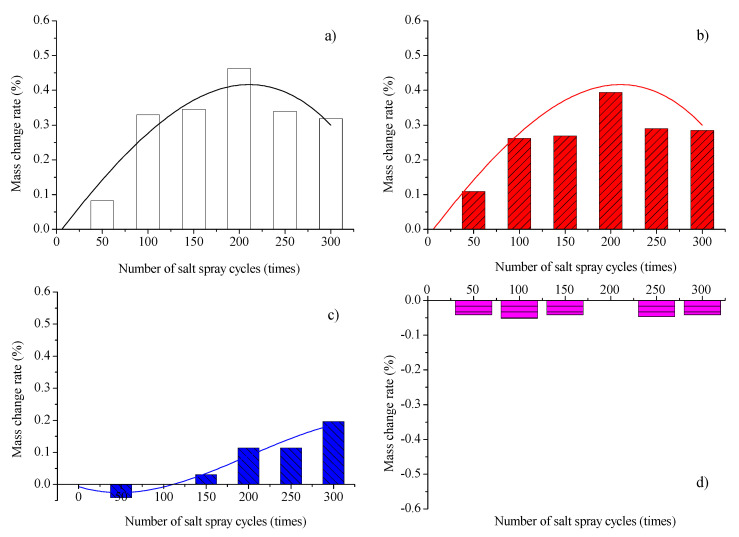
Change rate of mass; (**a**) Control RCC; (**b**) RCC-SS; (**c**) RCC-IOTS; (**d**) RCC-PUA.

The mass change rate of RCC under salt spray cycles is given in Figure 6. For both the reference RCC and RCC-SS, the mass change rate initially increases and then decreases as the number of salt spray cycles increases. Zhang et al. [2] also found a similar pattern of mass change. The maximum rate of mass increase occurs after 200 cycles of salt spray. The initial increase in the change rate is primarily attributed to increased salt permeation and crystallization. The crystals fill and cover the pores, resulting in an increase in concrete mass [2,39]. As the cycles progress, the decrease in the change rate is mainly caused by the intensified corrosive effect of salt in the salt spray, leading to localized delamination of the surface layer [2]. However, the extent of delamination at this stage is still smaller than the mass increase resulting from salt filling and crystallization in the concrete pores. Consequently, the mass change rate remains positive but exhibits a decreasing trend.

For RCC treated with IOTS, the mass loss rate of the specimens subjected to 50 cycles of salt spray is 0.03%. After 100 cycles of salt spray, the rate of mass change of the specimens first approaches zero and then increases with the number of salt spray cycles. At 300 cycles of salt spray, the mass increase rate is 0.15%, which is only about 50% of the control RCC subjected to the same number of salt spray cycles. For RCC-PUA, the rate of mass loss of the specimens remains at approximately 0.04% as the number of salt spray cycles increases.

Therefore, both RCC-IOTS and RCC-PUA surface coatings exhibit favorable resistance to salt spray corrosion when considering the criterion of mass change in a specimen under salt spray cycles.

### 3.3. Abrasion Resistance

The use of concrete in runway construction exposes it to rubbing, scraping, skidding, and sliding due to the impact loads from surface movement. These actions contribute to the deterioration of concrete surfaces. Surface fractures lead to a reduction in concrete thickness, resulting in a smoother surface and an increase in dust accumulation. These factors collectively weaken the concrete, posing a threat to flight safety. Therefore, it is imperative for concrete runways to possess adequate abrasion resistance—a property that shields the hardened concrete surface from wear caused by abrasive forces. Ensuring the abrasion resistance of concrete runways and pavements is crucial in preventing surfaces from becoming overly polished, thus maintaining optimal skid resistance.

**Figure 7:**
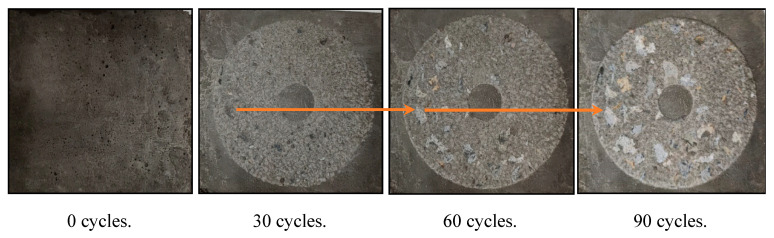
Surface change of control RCC.

The surface changes of the reference RCC with 300 cycles of salt spray are shown in Figure 7. It can be observed that with an increasing number of rotations of the grinding head, the surface wear of the hardened cement paste becomes more evident, and the area of exposed coarse aggregates in the specimen increases. The surface changes of the other RCC with surface coatings are found to be similar to those of the control RCC with 300 cycles of salt spray. Exposed aggregates are visible on the sample surfaces, signaling a decline in the efficacy of the surface treatment layers. Eventually, these layers are fully removed, indicating a complete loss of their protective effect [40].

**Figure 8:**
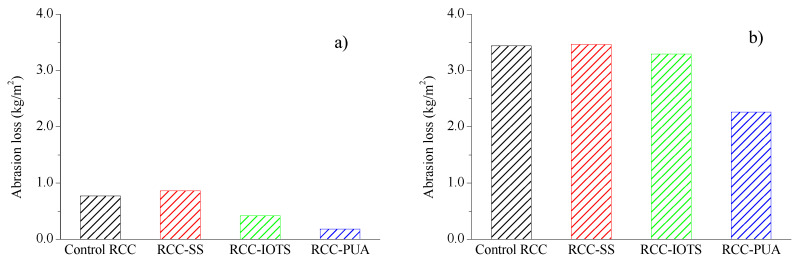
Abrasion loss. (**a**) 30 cycles; (**b**) 90 cycles.

The abrasion loss per unit area of RCC with 30 and 90 rotations of the grinding head are presented in Figure 8a,b, respectively. It is clear that the abrasion loss per unit area of reference RCC and RCC-SS is very close. However, Franzoni et al. [34] discovered that treatment with sodium silicate (SS) yielded the most effective performance in enhancing concrete’s surface abrasion resistance, attributed to the substantial thickness of the resulting external layer. The difference between the results of this study and those of Franzoni et al. [34] is mainly due to the thickness of the SS coating; a larger coating thickness tends to increase the wear resistance of the specimen, whereas in, this study, only 20% SS solution was sprayed on the specimen four times at 2 h intervals, which produced a thinner SS coating; therefore, the determination of the coating thickness is as critical as the selection of the coating material.

Both RCC-IOTS and RCC-PUA exhibit significantly lower abrasion losses per unit area. After 90 cycles of grinding head rotation, RCC-IOTS and RCC-PUA experienced abrasion losses of 3.29 kg/m^2^ and 2.26 kg/m^2^, respectively—indicating reductions of 4.4% and 34.3% compared to the control RCC. Wu et al. [40] also noted that PUA surface treatments led to a notable decrease in concrete mass loss, signifying enhanced resistance to debris flow abrasion. Baltazar et al. [41] observed that as long as the PUA protective layer remained intact on the concrete surface, it imparted excellent abrasion resistance. The findings in this study underscore that PUA treatment excels in both abrasion resistance and salt spray resistance.

### 3.4. Dynamic Elastic Modulus and Compressive Strength

**Figure 9:**
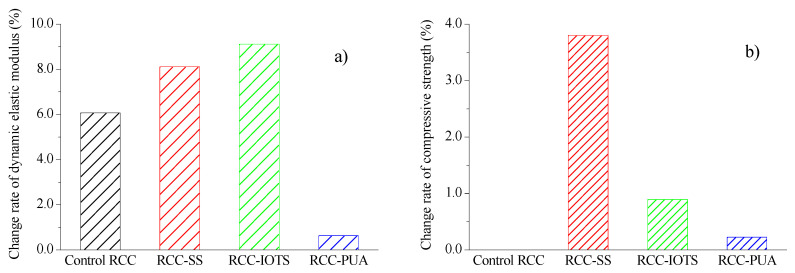
Change rate of dynamic elastic modulus and compressive strength. (**a**) dynamic elastic modulus, (**b**) compressive strength.

The percentage change in dynamic elastic modulus of RCC with 300 cycles of salt spray is shown in Figure 9a. It can be observed that all RCC samples experience varying degrees of increase in dynamic elastic modulus after the salt spray cycles. Zhang et al. [2] also reported similar findings when concrete specimens were subjected to salt spray cycles. RCC-IOTS showed the highest increase, reaching 9.1%, while RCC-PUA showed the lowest increase, at 0.6%. There are two possible reasons for the increase in the dynamic elastic modulus. Firstly, during the 300 salt spray cycles, the specimens undergo a 60-day period, enabling continued cement hydration and subsequent increase in the dynamic elastic modulus. Secondly, a large amount of salt penetrates into RCC specimens during the salt spray cycles. The salt crystallizes within the pores and fills some of them during the drying process, thereby increasing the compactness and reducing the porosity of RCC to some extent. Both factors contribute to the increase in the dynamic elastic modulus of RCC. The relatively minimal increase in the elastic modulus of RCC-PUA is attributed to the formation of a complete, smooth, and dense sealing layer on the specimen’s surface. Under such conditions, only the first mechanism mentioned above, related to cement hydration, significantly impacts the increase, while the second mechanism has minimal influence.

The relative change in the compressive strength of RCC with surface coatings after 300 cycles of salt spray is depicted in Figure 9b. It clearly shows that RCC-SS exhibits the highest compressive strength, followed by RCC-IOTS, while RCC-PUA shows the least improvement. The compressive strength of RCC-SS, RCC-IOTS, and RCC-PUA is 3.8%, 0.89%, and 0.22% higher than that of control RCC after 300 cycles of salt spray. These results demonstrate that all three surface coatings have enhanced the resistance of RCC to salt spray corrosion to varying degrees.

### 3.5. Impact Resistance

**Figure 10:**
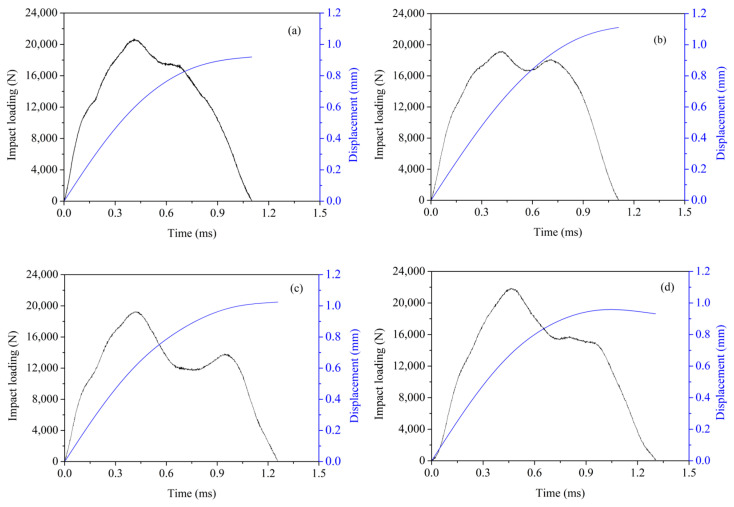
Impact test. (**a**) Control RCC; (**b**) RCC-SS; (**c**) RCC-IOTS; (**d**) RCC-PUA.

In the drop hammer impact test, each impact’s energy is controlled at 13 J, and continuous impact loads are applied until the fracture of the specimen occurs. Figure 10 illustrates the relationship between the impact force and time during the first impact after the specimens are subject to 300 cycles of salt spray. It is evident that different groups of RCC exhibit varying peak impact forces under approximately the same impact energy. RCC-PUA displays the highest peak force at 21.9 kN, indicating the highest surface hardness after the salt spray cycles. Wu et al. [40] observed that concrete treated with PUA exhibited a harder surface compared to non-coated concrete.

The reference RCC followed with a peak impact force of 20.7 kN, while RCC-SS and RCC-IOTS show similar peak impact forces at around 19.2 kN, with two peaks observed. The second peak is likely attributed to the loosening of the surface layer of the specimens due to corrosion. When the impact force is applied, the loose surface concrete becomes compacted and comes into contact with the non-corroded and harder concrete in the interior. In addition, some of the following factors may also cause a second wave peak [42,43]: (1) the presence of eccentricity or an overly sharp head of the hammer may result in multiple peaks during impact; (2) non-homogeneous concrete may experience multiple peaks of strain under impact, resulting in corresponding peaks of impact force (which is the case with the results of the present study); (3) the flatness of the impact surface affects the absorption and transfer of energy, which results in multiple peaks of impact force; (4) multiple peaks of impact force may also occur when only a portion of the hammer head contacts the concrete.

Table 4 presents the impact energy per impact, loss rate of the dynamic elastic modulus, and number of impacts until specimen fracture. It can be observed that the reference RCC fractures at the second impact, while other RCCs with different surface coatings fracture at the third impact. The impact energy before fracture is approximately 13 J for all specimens but decreases at the point of fracture. The total absorbed energy at fracture increases by 64.8%, 53.2%, and 50.1% for the different surface-coated RCC specimens. During the first impact, the control RCC exhibits the highest loss rate of the dynamic elastic modulus at 10.3%, followed by RCC-PUA at 2.6%. RCC-SS and RCC-IOTS show very low loss rates of dynamic elastic modulus at 0.4% and 0.2%, respectively. Overall, the surface coatings significantly improve the impact resistance of RCC under salt spray conditions. Wu et al. [40] used a drop-weight impact test to assess the impact resistance of concrete, noting no apparent failure and only changes in color on the PUA surface. The ductile behavior of PUA material contributed significantly to the impact resistance of concrete treated with PUA.

### 3.6. Microstructure and Pore Structures

**Figure 11:**
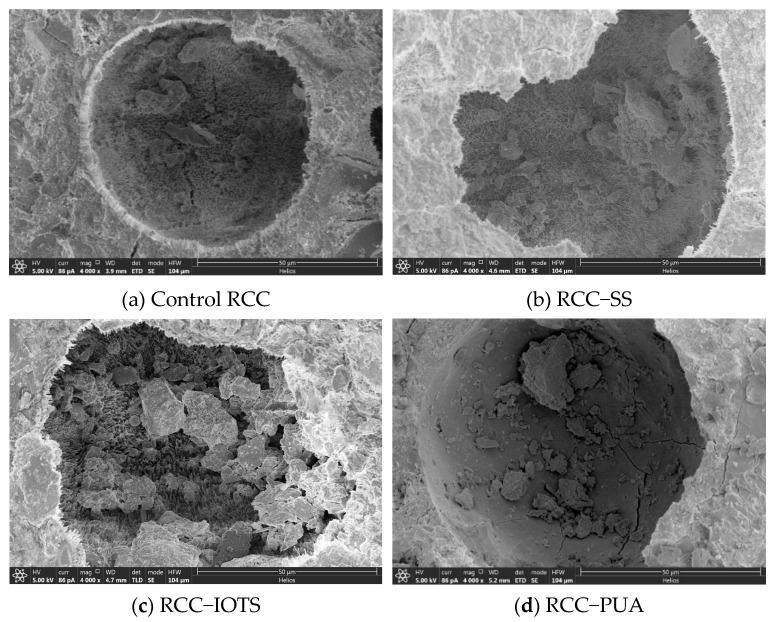
Microstructure of RCC.

Figure 11 presents the microstructures of RCC subjected to 300 cycles of salt spray. After the salt spray cycles, crystalline fillers are present in the surface pores of the control RCC, as well as in RCC-SS and RCC-IOTS. Although a thin protective film may form only on the pore wall, salt can still enter the pores of RCC-SS and RCC-IOTS, similar to the uncoated RCC, and crystallize during the drying process. Nonetheless, due to the presence of the protective coating, the direct contact between the salt and concrete layer is prevented until the protective coating is damaged. However, the PUA surface coating forms a complete and dense protective layer on the surface, effectively isolating the internal pores from salt penetration.

Elemental analysis of the fillers in the internal pores shows sodium (Na) contents of 4.6%, 3.4%, 2.8%, and 0.5% for the control RCC, RCC-SS, RCC-IOTS, and RCC-PUA, respectively. Furthermore, this study revealed that surface coatings, particularly PUA, can effectively deter the infiltration of sodium sulfate into concrete. After 300 salt spray cycles, concrete treated with PUA exhibited the lowest sodium (Na) content.

**Figure 12:**
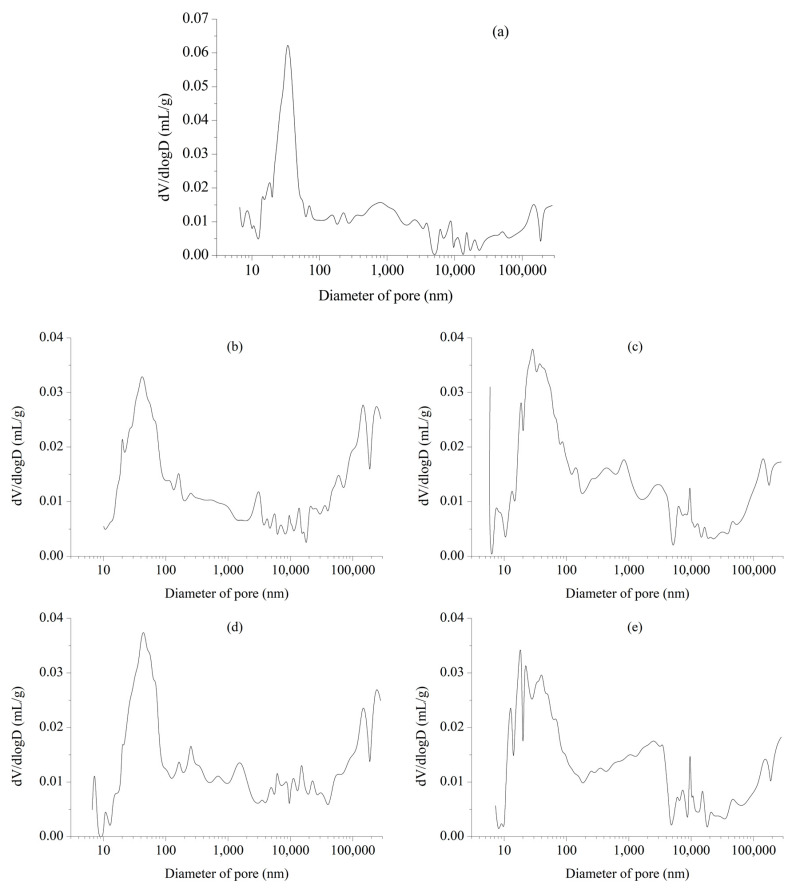
Pore size distribution of RCC. (**a**) RCC under standard conditions; (**b**) Control RCC; (**c**) RCC-SS; (**d**) RCC-IOTS; (**e**) RCC-PUA.

In addition, clear microcracks are observed in the internal and edge regions of the pores in the uncoated RCC, which indicates that the internal pores have suffered damage after 300 cycles of salt spray. Although the pores of RCC-SS and RCC-IOTS are filled with crystals and no significant microcracks are found, the changing trend of the pore size distribution (see Figure 12) indicates a movement towards larger pore sizes, suggesting some degree of damage after 300 cycles of salt spray for all three surface-coated RCCs.

### 3.7. Discussion

There are three main types of hydrophobic surface treatments: (1) surface coatings, which form a continuous film of varying thickness on the surface; (2) pore filling, which acts as a localized pore barrier; and (3) impregnation or pore lining, which involves lining the pores along the entire surface of the concrete.

The SS coating undergoes hydrolysis at room temperature, forming an interconnected network structure, as shown in the following reaction:

(3) $${\mathrm{Na}}_{2}O\cdot \mathrm{nSi}O_{2}+\left(2n+1\right)H_{2}O\to 2\mathrm{NaOH}+n{\mathrm{Si}(\mathrm{OH})}_{4}$$

(4) $${\mathrm{nSi}(\mathrm{OH})}_{4}\to {[\mathrm{Si}{\left(\mathrm{OH}\right)}_{4}]}_{n}\overset{-2nH_{2}O}{\to }{\left[-\mathrm{Si}-O-\mathrm{Si}-\right]}_{n}$$

The resulting thin film material adheres to the concrete surface, creating a separation between the concrete and its surrounding environment. However, due to the solubility of Na+ in water, the water glass-formed film generally exhibits moderate impermeability. Nevertheless, when the surface water glass penetrates into the interior of the concrete, it reacts with the cement hydration product Ca(OH)_2_, generating hydrated calcium silicate gel that fills the concrete pores, making it more compact and enhancing the durability of concrete [44,45]. Therefore, several properties of RCC-SS in this study were also improved, with a 22.8% reduction in water absorption, a 3.8% increase in compressive strength, and a 64.8% increase in impact energy absorption.

**Figure 13:**
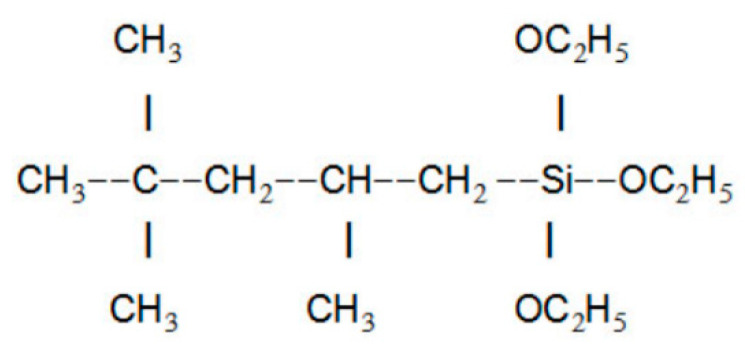
Chemical molecular structure of IOTS.

The chemical molecular structure of IOTS is shown in Figure 13. IOTS can impart excellent hydrophobicity to concrete surfaces without altering their surface microstructures.

This phenomenon is consistent with Wenzel’s theory, which suggests that rough mortar samples can be changed to hydrophobic surfaces after modification with low surface energy materials. This process involves the hydration of IOTS to form silanols (Si-OH), followed by the reaction of silanol with C-S-H, Ca(OH)_2_, ettringite, and quartz sand through -OH group reactions. Subsequently, the two -OH groups of IOTS form Si-O-Si bonds via condensation and release water in the process. As a result, a continuous self-assembled molecular film of IOTS is formed on the surface of the hydrated products. The presence of -CH_3_ and -CH_2_ groups in IOTS effectively reduces the surface energy of the cement matrix and significantly improves its hydrophobicity [46]. Similarly, several properties of RCC-IOTS in this study were significantly improved, with a 77.2% reduction in water absorption, a 4.4% reduction in the abrasion loss per unit area, and a 53.2% increase in impact energy absorption.

**Figure 14:**
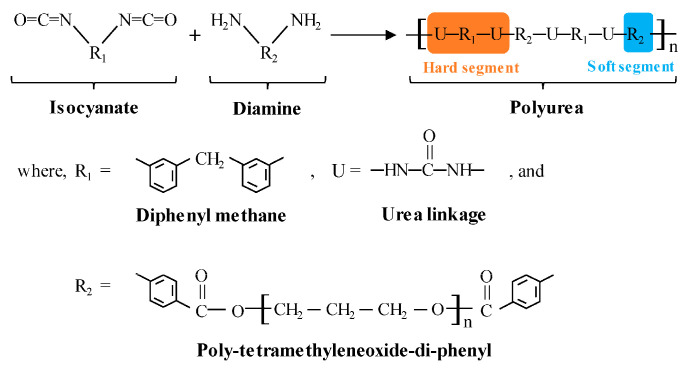
Chemical molecular structure of PUA [47].

PUA is a block polymer material, as given in Figure 14 [47]. It consists of hard segments and soft segments. The hard segments are uniformly distributed in the soft segment matrix at room temperature to form an interconnected network microstructure.

The strength of PUA primarily relies on its hard segments, while the elongation is determined by the soft segments. Due to its unique properties, PUA forms a seamless, leak-free membrane, making it highly suitable for enduring continuous ponding water conditions. Moreover, PUA coatings are renowned for their exceptional durability, offering remarkable elongation and tensile strength, making them an excellent choice for various surface coating applications [48,49]. Several properties of RCC-PUA in this study were significantly improved, with an 89.8% reduction in water absorption, a 34.3% reduction in the abrasion loss per unit area, and a 50.1% increase in impact energy absorption.

## 4. Conclusions

This study presented the deterioration of RCC both with and without surface coatings during salt spray cycles. The following conclusions can be drawn from the test results:

1. Prior to salt spray exposure, RCC-SS, RCC-IOTS, and RCC-PUA exhibited 24 h water absorption rates 22.8%, 77.2%, and 89.8% lower than those of the control RCC, respectively.
2. After 300 cycles of salt spray, the abrasion loss per unit area of RCC-SS, RCC-IOTS, and RCC-PUA is reduced by 0.3%, 4.4%, and 34.3%, respectively, compared to the control RCC.
3. The compressive strength of RCC-SS, RCC-IOTS, and RCC-PUA is higher by 3.8%, 0.89%, and 0.22%, and the total absorbed energy at fracture is 64.8%, 53.2%, and 50.1% higher than that of control RCC, respectively.
4. Crystalline fillers are found in the pores of control RCC, RCC-SS, and RCC-IOTS, excluding RCC-PUA. However, the volume percentage of small pores in all RCCs decreases, while the volume percentage of large pores increases.

## Figures and Tables

**Table 1:** Chemical compositions of cement (%).

SiO_2_	Al_2_O_3_	Fe_2_O_3_	CaO	MgO	SO_3_	Na_2_O	K_2_O	TiO_2_
23.1	7.1	3.67	57.59	2.18	2.65	0.18	0.72	0.34

**Table 2:** Mix proportions of RCC (kg/m^3^).

Cement	Water	Fine Aggregate	Coarse Aggregate	Water-Reducing Agent
315	109	895	1207	8.7

**Table 3:** Surface-coating materials.

	Solution Concentration	Surface Treatment Age
Sodium silicate (SS)	20%	7 day
Isooctyl triethoxy silane (IOTS)	99%	28 day
Polyurea (PUA)	80%	28 day

**Table 4:** Impact resistance.

	RCC		RCC-SS			RCC-IOTS			RCC-PUA		
Impact number (times)	1	2	1	2	3	1	2	3	1	2	3
Impact energy (J)	12.9	9.1	12.9	13.0	10.4	13.0	12.9	7.8	13.0	13.1	6.9
Loss rate DEM * (%)	10.3	/	0.4	3.2	/	0.2	61.5	/	2.6	51.3	/

* DEM is dynamic modulus of elasticity.

## Data Availability

Data are contained within the article.
